# Spatial and Temporal Control of Cavitation Allows High *In Vitro* Transfection Efficiency in the Absence of Transfection Reagents or Contrast Agents

**DOI:** 10.1371/journal.pone.0134247

**Published:** 2015-08-14

**Authors:** Kamel Chettab, Stéphanie Roux, Doriane Mathé, Emeline Cros-Perrial, Maxime Lafond, Cyril Lafon, Charles Dumontet, Jean-Louis Mestas

**Affiliations:** 1 Université de Lyon, 69000, Lyon, France; 2 Université de Lyon 1, 69000, Lyon, France; 3 INSERM U1052, Centre de Recherche en Cancérologie de Lyon, 69008, Lyon, France; 4 CNRS UMR 5286, Centre de Recherche en Cancérologie de Lyon, 69008, Lyon, France; 5 Caviskills SAS, Vaulx-en-Velin, France; 6 Inserm, U1032, LabTau, Lyon, F-69003, France; University of Hawaii, UNITED STATES

## Abstract

Sonoporation using low-frequency high-pressure ultrasound (US) is a non-viral approach for *in vitro* and *in vivo* gene delivery. In this study, we developed a new sonoporation device designed for spatial and temporal control of ultrasound cavitation. The regulation system incorporated in the device allowed a real-time control of the cavitation level during sonoporation. This device was evaluated for the *in vitro* transfection efficiency of a plasmid coding for Green Fluorescent Protein (pEGFP-C1) in adherent and non-adherent cell lines. The transfection efficiency of the device was compared to those observed with lipofection and nucleofection methods. In both adherent and non-adherent cell lines, the sonoporation device allowed high rate of transfection of pEGFP-C1 (40–80%), as determined by flow cytometry analysis of GFP expression, along with a low rate of mortality assessed by propidium iodide staining. The transfection efficiency and toxicity of sonoporation on the non-adherent cell lines Jurkat and K562 were similar to those of nucleofection, while these two cell lines were resistant to transfection by lipofection. Moreover, sonoporation was used to produce three stably transfected human lymphoma and leukemia lines. Significant transfection efficiency was also observed in two fresh samples of human acute myeloid leukemia cells. In conclusion, we developed a user-friendly and cost-effective ultrasound device, well adapted for routine *in vitro* high-yield transfection experiments and which does not require the use of any transfection reagent or gas micro-bubbles.

## Introduction

Transfection is a widely used tool for the analysis of the role of a target gene, determination of its function and identification of regulatory sequences. Different methods are currently available for the transfection of nucleic acid transfection into eukaryotic cells. There are two major types of strategies for gene delivery: viral and non-viral methods, the latter including chemical and physical methods. Viral vectors provide the most efficient means for *in vivo* and *in vi*tro gene delivery. However, the use of viral vectors carries significant biological risks such as uncontrolled activation of immunogenic response, carcinogenesis, broad tropism and difficulty of vector production [1–4]. Furthermore, although highly efficient *in vitro*, viral delivery systems are expensive, time-consuming, and require laboratories to comply with stringent safety regulations. On the other hand, chemical methods using natural or synthetic carriers to deliver genes into cells are safe and effective for transfection of adherent cell lines but suffer from their low efficiency in the case of non-adherent cell lines as well as *in vivo* [5–7]. Thus the development of more efficient, cheaper and safe gene delivery methods is an important issue for gene therapy research.

Physical methods for gene delivery such as micro-injection, electroporation, gene gun and ultrasound-mediated methods have been the object of considerable research during the last decade. Physical methods aim to increase the permeability of the cell membrane, allowing genetic material and therapeutic molecules to enter into the cell [8–10]. A number of studies have analyzed ultrasound as a tool to internalize DNA, siRNA, antibodies, and chemotherapeutic drugs both *in vitro* and *in vivo* [11–14, 15–17]. Ultrasound-induced biological effects are commonly assumed to be caused by acoustic cavitation [15, 18–19] which results in the creation of transient holes in the cell membrane allowing the intracellular penetration of nucleic acids or drugs [11–14, 20]. Conversely ultrasound application generates localized fluid flow, shear stress, and other mechanical or physical impacts which may lead to irreversible cell damage and cell death under certain conditions [19, 21–22].

There is a considerable heterogeneity in the literature concerning sonoporation devices and procedures, leading to marked differences in transfection efficiency [10, 23–25]. Another limitation is the fact that most devices are not user-friendly and therefore not adapted for routine use in research laboratories [17, 25–26]. Some investigators have obtained improved transfection efficiency and chemotherapeutic drug delivery using ultrasound by combining this approach with contrast agents [23–29]. This combination approach is relatively complicated to optimize, as well as time- and money-consuming.

Here we report the development of a new ultrasound device dedicated to efficient transfection of adherent and non-adherent cells, including both transient transfection and the production of long-term stable transfectants. The development of this device was mainly focused on the spatial and temporal control of ultrasound cavitation allowing reliable and high level transfection efficiency in both adherent and non-adherent cells. This device is easy to use, well adapted for routine transfection for a low unit cost and allows a high transfection rate which does not require the use of transfection reagents or contrast agents.

## Materials and Methods

### 2.1 Cell and culture conditions

Non-adherent cell lines, including the human follicular lymphoma cell line RL, the T-lymphoblastoid cell line Jurkat, the human chronic myelogenous leukemia cell line K562, the human promyelocytic leukemia cell line HL60 as well as the adherent human breast tumor cell line BT474 were cultured in RPMI 1640 medium. The human lung adenocarcinoma epithelial cell line A549 was cultured in Dulbecco’s modified Eagle medium (DMEM). Both media were supplemented with 10% heat inactivated fetal calf serum (FCS), 200 UI/ml of penicillin and 200 μg/ml of streptomycin. The cells were maintained at 37°C with 5% CO_2_. RL, Jurkat, HL60, and K562 cell line were obtained from the American Type Culture Collection (ATCC, Manassas, VA, USA). BT474 and A549 cell lines were purchased from DSMZ (Leinik Institute DSMZ-German collection of microorganisms and cell cultures, Braunschweig, Germany). All reagents were purchased from life technologies (Rockville, MA, USA). Using MycoAlert kit (Lonza, Basel, Switzerland) all cell lines were tested mycoplasma-negative. Fresh Acute Myeloid Leukemia (AML) cells were obtained from two patients with signed informed consent and approval by the Lyon Ethics Committee. Cells were isolated from peripheral blood mononuclear cells by density gradient centrifugation using Histopaque (Pancoll human, PAN Biotech, Aidenbach, Germany). Briefly, blood was diluted in Phosphate Buffered Saline (PBS) then layered over Histopaque and was centrifuged at 300g for 20 min at room temperature. The gradient interface was harvested and was diluted 3-fold with PBS. The cell suspension was washed 3 times by repeated centrifugation at 300g for 10 min and resuspended in complete RPMI 1640 media. Cell viability was evaluated by trypan blue dye.

### 2.2 Plasmid DNA preparation

A green fluorescent protein (GFP) gene expression vector driven by a cytomegalovirus promoter, peGFP-C1 (BD Biosciences, Clontech, CA, USA), carrying the neomycin resistance gene allowing the isolation of drug-resistant clones, was used as a marker for tracking gene transfection and expression. Expression vectors coding for shRNA against cN-II (pScN-II) and control sequence (pScont) were generously provided by Dr. Jordheim [30]. Plasmid DNA (pDNA) was amplified and purified using QIAprep Midiprep Kit (QIAGEN, CA, USA) according to the manufacturer’s protocol and dissolved in TE buffer (10 mM Tris-HCl, 1 mM ethylene-diaminetetra-acetic acid) at a concentration of 1 mg/ml. Plasmid concentration was measured using NanoDrop spectrophotometer (Thermo Fisher Scientific, Wilmington, USA) at 260 nm and purity was checked by measuring 260/280 and 260/230 ratios.

### 2.3 Ultrasonic set-up and cavitation control

**Fig 1:**
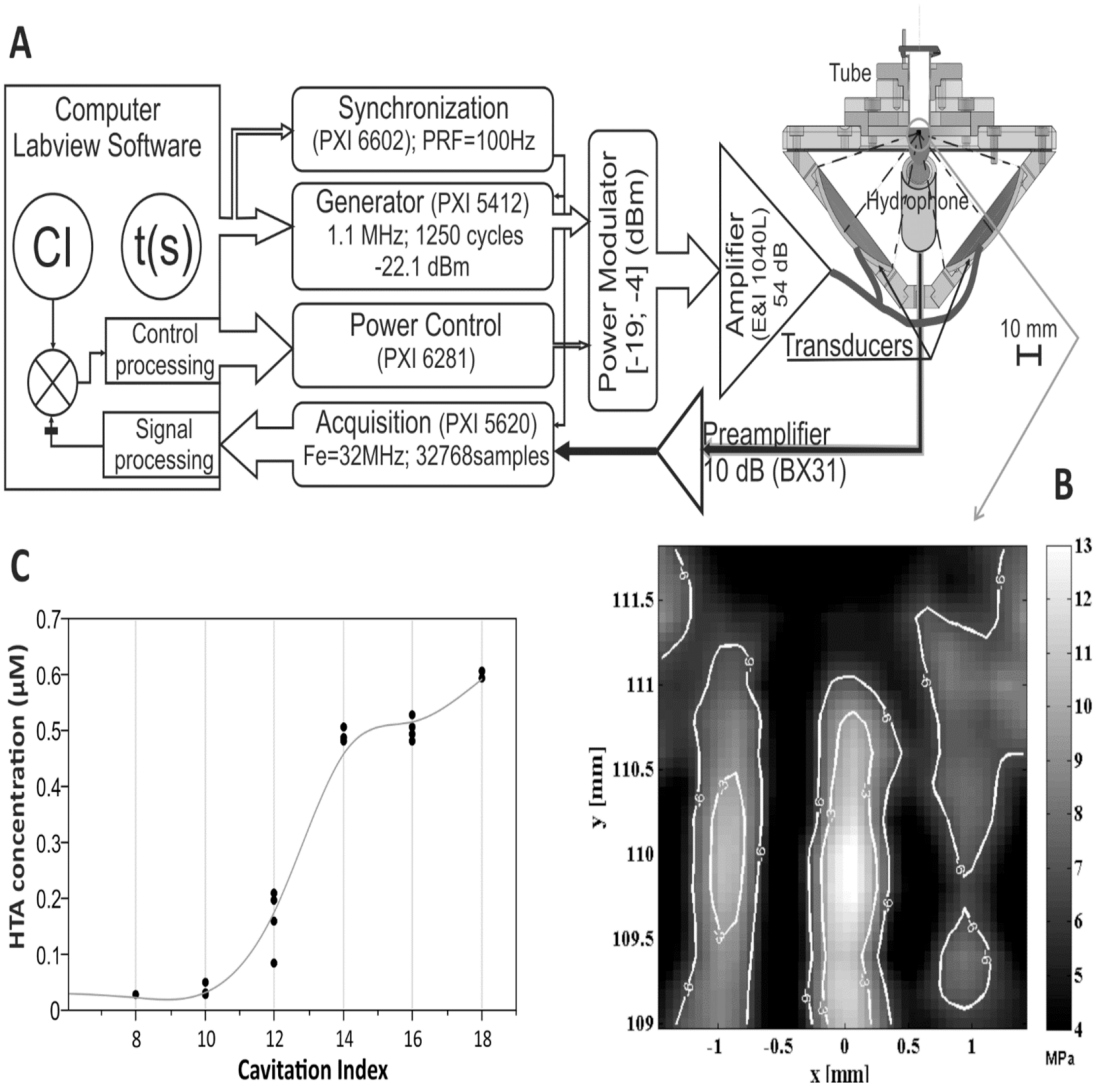
Experimental setup, cavitation control and pressure application. (A) Experimental setup. (B) Pressure field (dB) at the focal point (C) Relationship between the measured fluorescence levels in 550 μL of terephthalic acid medium (20 mM) and the cavitation index (sonication time: 60 seconds).

The ultrasonic set-up was based on two focused transducers PZ28 (Ferroperm, Kvistgaard, Denmark), operated at a frequency of 1.1 MHz. The diameter and the radius of curvature of these transducers was 50 mm. The transducers were positioned in a confocal manner with a 90° angle between the acoustic axes (Fig 1A). This set-up has several advantages such as the reduction of nonlinear propagation of the acoustic beams providing an increased pressure in the focal zone. The pressure field under free conditions presents a pattern of pressure nodes and antinodes due to the interference between the two ultrasound beams. The interference pattern traps the cavitation cloud, allowing the bubbles to reach the inertial cavitation threshold more easily [31]. Thus, the shape of the bubble cloud is approximately the shape of the focal volume. In a free field, the dimensions of the focal volume are 3x2x2 mm (length x width x elevation).

For transfection, longer pulses are applied to the culture medium (Pulse Repetition Frequency PRF: 100Hz; Duty cycle DC: 25%) confined in 2 ml tubes (Eppendorf, Hambourg, Germany). While this configuration results in multiple reflections inside the tube, the peak pressure remained at the same point, approximately 2 mm below the air/medium interface. Cavitation could be easily initiated with no more than 4 MPa negative pressure.

**Fig 2:**
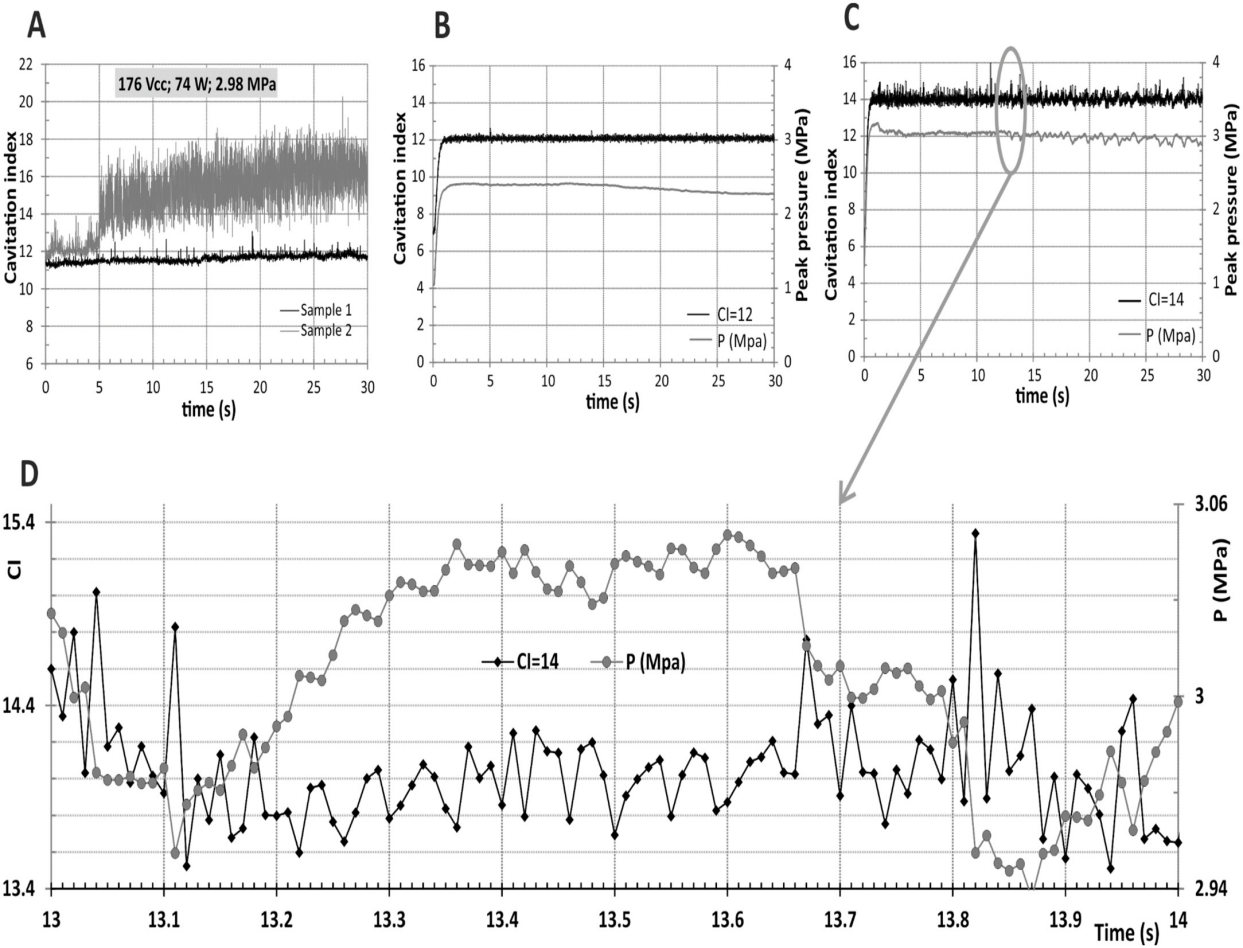
Inertial cavitation characterization and control. (A) CI responses using cavitation control process without feedback loop power (constant peak electric power applied: 74 W; corresponding peak pressure: ~3MPa). (B) and (C), 30 seconds sonication in 650 μL of water in a 2mL tube. (D) Zoom of Fig 2C from 13 to 14 seconds. The power was controlled to maintain CI at values of 12 and 14, respectively. The peak pressure was calculated from the monitored electrical power.

The focal pressure varies strictly with the electrical power delivered to the transducers. Cavitation, the phenomena of interest for transfection, is a very stochastic process by nature. Instead of operating at a constant pressure (Fig 2A) the electrical power was adjusted in real time during experiments thanks to the measurement of the inertial cavitation activity (Fig 2B–2D) by synchronizing the generator and the acquisition signal using a NI-PXI6602 card (National Instruments, Austin, Texas). The electrical signal (PRF 100Hz, DC 25%) is first emitted by the generator (NI-PXI 5412). It can then be modulated with a custom-made electronic board driven by a NI-PXI 6281 card, from -24 to -19 dBm depending on the instructions from the controlling software. The modulated signal is then amplified by a RF power amplifier (E&I 1040L Electronics & innovation Ltd, Rochester, New York; 400W, 54dB) and applied to the transducers. The electrical peak power ranges between 3 and 100 W corresponding to a peak pressure up to 8 MPa in water and ranging from 0.4 to 3.5 MPa inside the tube. The 650 μL sample (contained in a 2 ml tube, internal diameter 9.5mm) is placed in order for the focal point to be at 2 mm from the air/medium interface (Fig 1A). The surrounding/coupling media between the transducers and the tube is degassed water (concentration of dissolved O_2_ less than 3 mg/L) in order to avoid cavitation outside the sonicated sample. As the beam propagates, the strong pressure field (Fig 1B) at the focus point initiates the cavitation cloud. The broadband noise emitted by the collapsing bubbles during the inertial regimen of cavitation is recorded by a broadband hydrophone aimed toward the sample in the water tank (Fig 1A). This signal is pre-amplified by 10 dB (NF Electronic Instruments BX31), digitized (NI-PXI 5640 Analog to Digital Converter 14 bits; sampling frequency: 32 MHz) and 2^15^ data points (~1ms temporal signal) are saved temporarily on the computer. The cavitation index (CI) is calculated as follows:

$$CI\;(dB)\;=\;\frac{1}{\left(\beta -\alpha \right)}∙\sum_{i\;=\;\alpha }^{\beta }Y\left(f_{i}\right)-\;Ref$$

$$Ref\;=\;\frac{1}{\left(\beta -\alpha \right)}∙\sum_{i\;=\;\alpha }^{\beta }Y\left(f_{i}\right)\approx -93\;dB\;for\;pressure\;=\;0$$

$$Y_{n}\left(f\right)\;=\;\frac{N-1}{N}∙Y_{n-1}\left(f\right)+\frac{1}{N}∙X_{n}\left(f\right)\;⋰N\;=\;8$$

$$Y\left(f\right)\;=\;{\text{log}}_{10}\left[Y_{N}\left(f\right)\right]$$

δF = 7.8 kHz; α∙δF≅0.1 MHz; β∙δF≅7.1 MHz

(β-α) = 896

The acquired hydrophone signal is “divided” into 8 consecutive signals. A Fast Fourier Transform (FFT) is computed for each of them.

*X*_*n*_(*f*) is the FFT of the nth signal (1≤n≤8).

*Y*_*N*_(*f*) is a weighted average of these 8 FFTs.

The average of the spectral values (in dB) between 0.1 and 7.1 MHz (896 values) to which a reference value corresponding to the electronic noise (~-93 dB) is removed. The value corresponding to the electronic noise is obtained when the power amplifier is inhibited. CI is calculated for each ultrasonic pulse every 10 ms and is used to characterize the increase in white noise induced by the collapse of the bubble. The contribution of the signal harmonics in the CI computation is small compared to white noise [32].

Depending on the recorded level and the user's instruction of CI, the developed LabVIEW application (National Instruments) controls the power modulator and the output level is rectified in order to fit the CI instruction for the next burst. In order to demonstrate that the set-up is able to maintain a constant inertial cavitation activity for every burst each 10 ms, experiments were performed with a chemical dosimeter of inertial cavitation.

Acoustic cavitation generates free radicals from the breakdown of water and other molecules. When water is sonicated, OH radicals are formed as a result of water thermolysis. The initial step of water decomposition is the production of hydroxyl and hydrogen radicals. It has been shown that terephthalic acid (TA) [benzene-1, 4-dicarboxylic acid] is suitable for detecting and quantifying free hydroxyl radicals generated by the collapse of cavitation bubbles obtained with ultrasound irradiation. During this process, the TA solution (20 mM of TA in PBS) as a dosimetric solution reacts with a hydroxyl radical generated through water sonolysis. Therefore, 2-hydroxyterephthalic acid (HTA) is produced which can be detected using spectroscopy with excitation and emission wavelengths of 318 and 426 nm, respectively [33].

### 2.4 The Effect of ultrasound on plasmid integrity

To examine the effect of ultrasound on plasmid integrity, 2 ml tubes containing 10 μg of pEGFP-C1 in 650 μl of Opti-MEM (Life Technologies, Rockville, MA) were separately exposed for 5, 10, 20, 30, 60 and 80 seconds at a CI of 12. In parallel, we also tested different CI values (10, 12, 14 and 16) for 30 seconds. After ultrasound exposure the integrity of each plasmid sample was examined by agarose-ethidium bromide electrophoresis. The result was compared with control pEGFP-C1 which had not undergone ultrasonic exposure.

### 2.5 Sonoporation procedures

Forty-eight hours prior to transfection, non-adherent cells were seeded at a concentration of 0.2–0.4.10^6^ cells/ml, in order to achieve 0.5–0.8.10^6^ cells/ml on the day of sonoporation. Adherent cells were plated at 2.10^6^ cells per 75 cm^2^ in order to achieve approximately 70% confluence after 48 hours. Prior to ultrasound exposition, non-adherent or trypsinized adherent cell lines were harvested and centrifuged (i.e., 10 min, 100g) then washed twice with Opti-MEM to remove serum traces, a step which significantly improves transfection efficiency [29]. 550 μl or 650 μl of cell suspensions (2.10^6^ or 10.10^6^ cells/ml in Opti-MEM were placed in 2 ml tube and pEGFP-C1 plasmid was added to cell suspensions at the indicated concentration. After ultrasound application, cell suspensions were placed in complete medium in a 6-well cell culture plate and incubated at 37°C in humidified atmosphere with 5% CO_2_ incubator for 24 hours.

### 2.6 Nucleofection and lipofection transfection assays

We performed a study to compare the transfection efficiency and the cell viability of sonoporation with those of two commercially available transfection methods: nucleofection and lipofection. This study was performed on two non-adherent cell lines, Jurkat and K562. For nucleofection transfection, the procedure was performed according to the manufacturer’s recommendations (Lonza, Basel, Switzerland; previously, Amaxa Biosystems). Briefly, Jurkat and K562 cells were pelleted by centrifugation at 90g and 200g for 10 minutes, respectively, and resuspended at a density of 10.10^6^ cells/ml in 100 μl Nucleofactor Solution V (Cell Line Solution V Nucleofector Lonza) at room-temperature. Two micrograms of pEGFP-C1 vector were added to the cell suspension. The cell / DNA mixtures were nucleoporated in 1 cm transfection cuvettes according to a predefined program on the Nucleofactor. Twenty-four hours post-nucleofection the transfection efficiency and cell viability were evaluated by flow cytometry after propidium iodide (PI) staining (Roche Diagnostics, Mannheim, Germany). Lipofection using Lipofectamine 2000 Transfection Reagent (Life Technologies, Rockville, MA) was conducted on the Jurkat and K562 cell lines according to the manufacturer’s instructions.

### 2.7 Analysis of transfection efficiency and cell viability

In this study the plasmid peGFP-C1, encoding GFP under the control of a CMV promoter was employed to monitor transfection. Twenty-four hours after sonoporation of peGFP-C1 vector, GFP-positive cells were observed using an Olympus IX50 microscope at a excitation wavelength of 488 nm. The transfection efficiency and cell viability were monitored using a flow cytometer (fluorescence-activated cell sorter) (FACS) (LSR II, BD Biosciences, CA, USA). Adherent cells were harvested by trypsinisation as well as non-adherent cells present in the culture medium. The cell suspension was centrifuged (10 min, 100g) then washed twice in PBS. In order to assess cell viability, PI was added to cells for 10 min according to the manufacturer's recommendations prior to FACS analysis. During FACS analysis the gate was drawn to exclude debris. Results were expressed as a percentage of GFP positive cells using BD Biosciences FACS Diva software. This percentage was calculated on the basis of the viable cells.

### 2.8 Statistical analysis

To determine reproducibility, three independent experiments were conducted (each performed at least in triplicate). All results are reported and displayed as mean ± standard deviation. Significance tests were performed using a non-parametric Wilcoxon test (JMP, SAS Institute Inc., Cary, NC). Significance was defined as p < 0.05.

## Results

### 3.1 Inertial cavitation characterization and control

The inertial cavitation was characterized by a chemical dosimeter TA for OH radicals. The resulting hydroxyterephthalate fluorescence is adequately correlated with the cavitation index (Fig 1C) which confirms the presence of the inertial cavitation (irreversible bubble collapses in the exposed medium). Fig 2A presents CI responses obtained without feedback loop (peak electric power setting 74 W; corresponding peak pressure: ~3MPa). They could be compared with those obtained with the control process for setting CI = 12 (Fig 2B) and CI = 14 (Fig 2C and 2D). With the control process CI reaches the target value in less than 0.6 seconds. For the two CI settings 12 and 14, the corresponding observed mean values were respectively 12.0±0.1 and 14.0±0.2 for the flat part of the responses. For comparison the measured CI for samples 1 and 2 induced by the same electric power were respectively 11.6±0.2 and 15.0±1.8. Our cavitation process remained stable over time. Fig 2B–2D show the controlled pressures obtained to maintain the desired CI values (12 and 14) and the corresponding application peak pressure calculated from the monitored electrical power. Fig 2D is a zoom of Fig 2C and presents CI values computed from the measurement and pressure values calculated from controlled processing to be applied on consecutive pulses every 10 ms.

### 3.2 Ultrasound exposure time and intensity did not affect plasmid integrity

**Fig 3:**
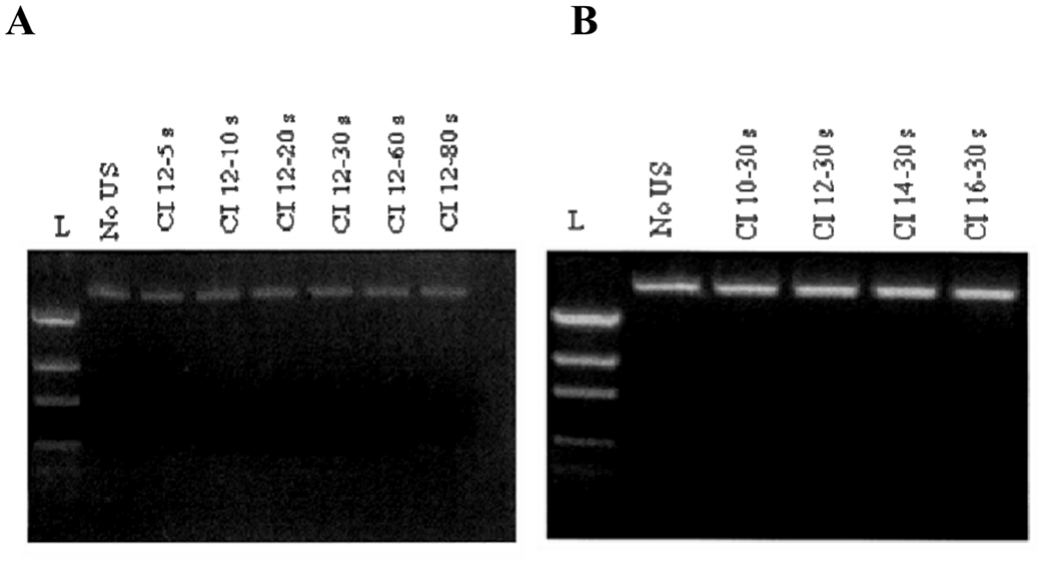
Effect of ultrasound on plasmid integrity. Agarose gel electrophoresis of peGF-C1 plasmid. (A) Ultrasound exposure for 5–80 seconds at CI level 12 did not affect plasmid integrity. (B) Plasmid exposure to different CI levels (10, 12, 14 and 16) for 30 seconds did not damage the plasmid integrity. L, Low DNA Ladder (Life Technologies, Rockville, MA, USA). The size and length of bands were the same between exposed and non-exposed plasmids, indicating that ultrasound irradiation does not affect plasmid integrity.

Maintaining plasmid integrity is an important issue when considering transfection by sonoporation. To examine whether ultrasound affects plasmid integrity, pEGFP-C1 was exposed to ultrasound either at a constant CI of 12 for a duration ranging from 0 to 80 seconds or at different CI values (10, 12, 14 and 16) for a constant time of exposure of 30 seconds. Contrary to the results published by Y. Song *et al*.[34] and in agreement with the results of L. Lin *et al*. [35], we found that plasmid integrity was not affected even at the highest CI level (CI 16/30 seconds), as assessed by gel electrophoresis (Fig 3).

### 3.3 Setting optimal sonoporation parameters

**Fig 4:**
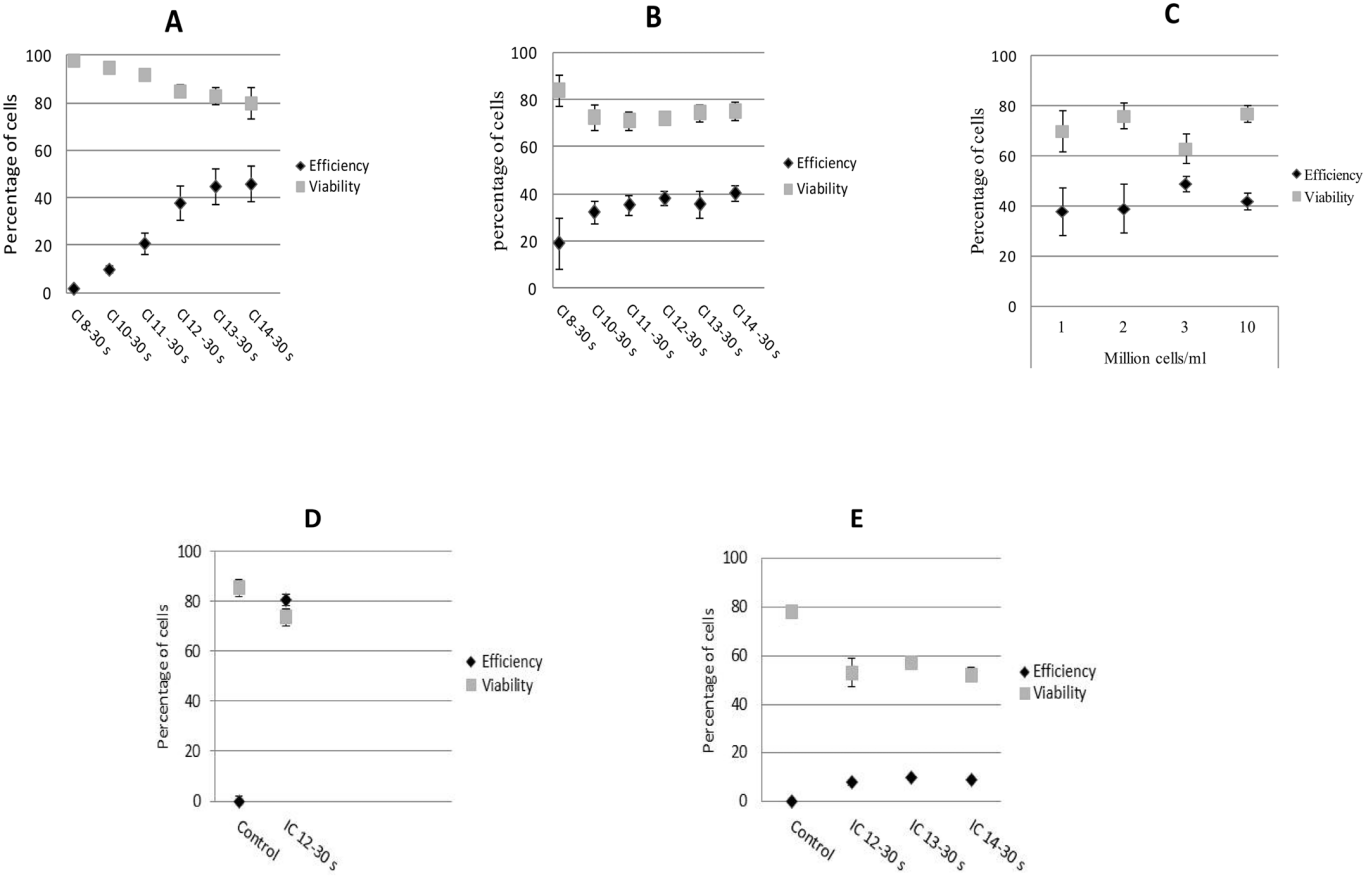
Influence of CI level and cell density on transfection efficiency and cell viability. Twenty-four hours post-sonoporation, the percentages of GFP-positive cells and levels of cytotoxicity were assessed by flow cytometry and PI staining. Two non-adherent cell lines Jurkat (A) and K562 (B) were exposed to CI values ranging between 8 and 14 during 30 seconds. The sonoporation with peGFP-C1 was performed in a 2 ml Eppendorf tube containing 10 μg of pEGFP-C1 in 550 μl of Opti-MEM medium. The cell concentration was set to 2.10^6^ cells/ml. (C) Transfection of 10 μg peGFP-C1 in Jurkat cells at four different cell densities (1.10^6^, 2.10^6^, 3.10^6^, and 10.10^6^ cells/ml). Percentage of GFP-positive cells and dead cells were determined at different cell densities using CI of 12 for 30 seconds. (D) Transfection efficiency and cell viability of the A549 cell line (10 μg peGFP-C1, 10.10^6^ cells/ml, and CI 12–30 seconds). (E) Transfection efficiency and cell viability of fresh AML cells. The error bars represent the mean (± standard deviation, SD) of triplicates and two measurements for Figs A-D and E, respectively.

In order to determine the effect of CI level on transfection efficiency, two non-adherent cell lines, Jurkat and K562, were exposed for 30 seconds at CI values ranging from 8 to 14. Twenty-four hours following ultrasound exposure, the transfection efficiency and cell viability were evaluated as described in Material and Methods. The results presented in Fig 4A and 4B confirm our previous data [11] and the results published by Lo CW *et al*. [17] showing that for non-adherent cell lines the transfection efficiency is proportional to the level of CI. Moreover, our results indicate that a CI value of 12 for 30 seconds insures a good compromise between transfection rate and cell viability. When applying CI levels greater than 14 we observed significant levels of cell mortality (data not shown). To investigate if cell density could affect overall transfection efficiency, we tested four Jurkat cell concentrations, 1.10^6^, 2.10^6^, 3.10^6^ and 10.10^6^ cells/ml. Flow cytometry analysis of GFP positive cells and cell viability (Fig 4C) demonstrated that the cell density did not influence transfection efficiency. However, a slight impact on technical reproducibility was observed with densities lower than 10.10^6^ cells/ml. Sonoporation of the lung epithelial cell line A549 under the same conditions, i.e., using the same cell and plasmid concentration as well as the same program, gave a transfection efficiency of 80% and 75% of cell viability (Fig 4D).

Fresh AML cells at a concentration of 10.10^6^ cells/ml were exposed for 30 seconds at three CI values (12, 13 and 14). Fig 4E shows a transfection efficiency of 10% for a CI value of 13 and 57% cell viability in comparison to 78% of cell viability observed in unexposed cells. These results are very encouraging and improvement is likely to be achieved by varying cell and plasmid concentrations. Transfection efficiency is comparable to that obtained in a recent study using nucleofection for gene transfer into primary human CD4^+^ T-cells [36]. Moreover cell viability assessed 24 hours post-sonoporation was higher than that obtained after DNA nucleofection [36].

### 3.4 Biological reproducibility of the device

**Fig 5:**
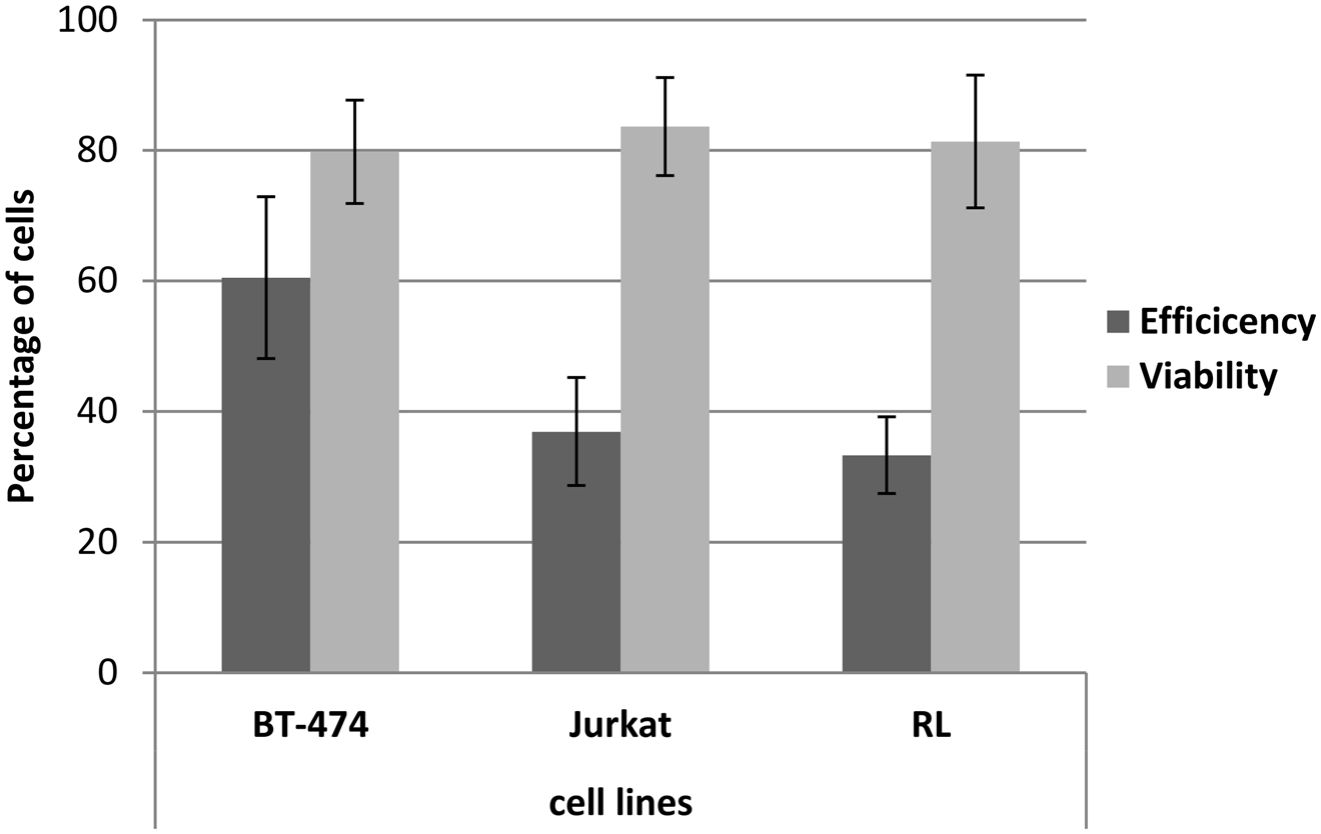
Biological reproducibility of the device. Assays were conducted on two non-adherent cell lines cell lines (RL and Jurkat) and one adherent cell line (BT474). Sonoporation was performed using the optimal parameters (10.10^6^ cells/ml, IC of 12 for 30 seconds and 10 μg of pEGFP-C1). Twenty-four hours post-transfection cells were collected and analyzed for transfection efficiency (% GFP+ cells) and for viability (% of cells excluding PI). All experiments were performed at least in triplicate and repeated independently three times for each cell line. Statistical analysis using a non-parametric Wilcoxon test demonstrated no significant difference in transfection efficiency and in cell viability.

To test the biological reproducibility of the device, sonoporation of the adherent cell line (BT474) and two non-adherent cell lines (RL and Jurkat) was conducted. Sonoporation was performed at a cell concentration of 10.10^6^ cells/ml using the optimal exposure conditions as described previously (CI of 12 for 30 seconds and 10 μg of pEGFP-C1). Cell viability was determined using PI staining. Results in Fig 5 show a high transfection efficiency and high cell viability in hard-to-transfect non-adherent cell lines. A non-parametric Wilcoxon test was used to study the reproducibility between three independent experiments which were performed in triplicates for each cell line. Statistical analysis showed no significant difference in transfection efficiency and in cell viability, providing evidence that support the reproducibility of the developed device.

### 3.5 Comparison of the transfection efficacy of pEGFP-C1 by sonoporation, nucleofection and lipofection

**Fig 6:**
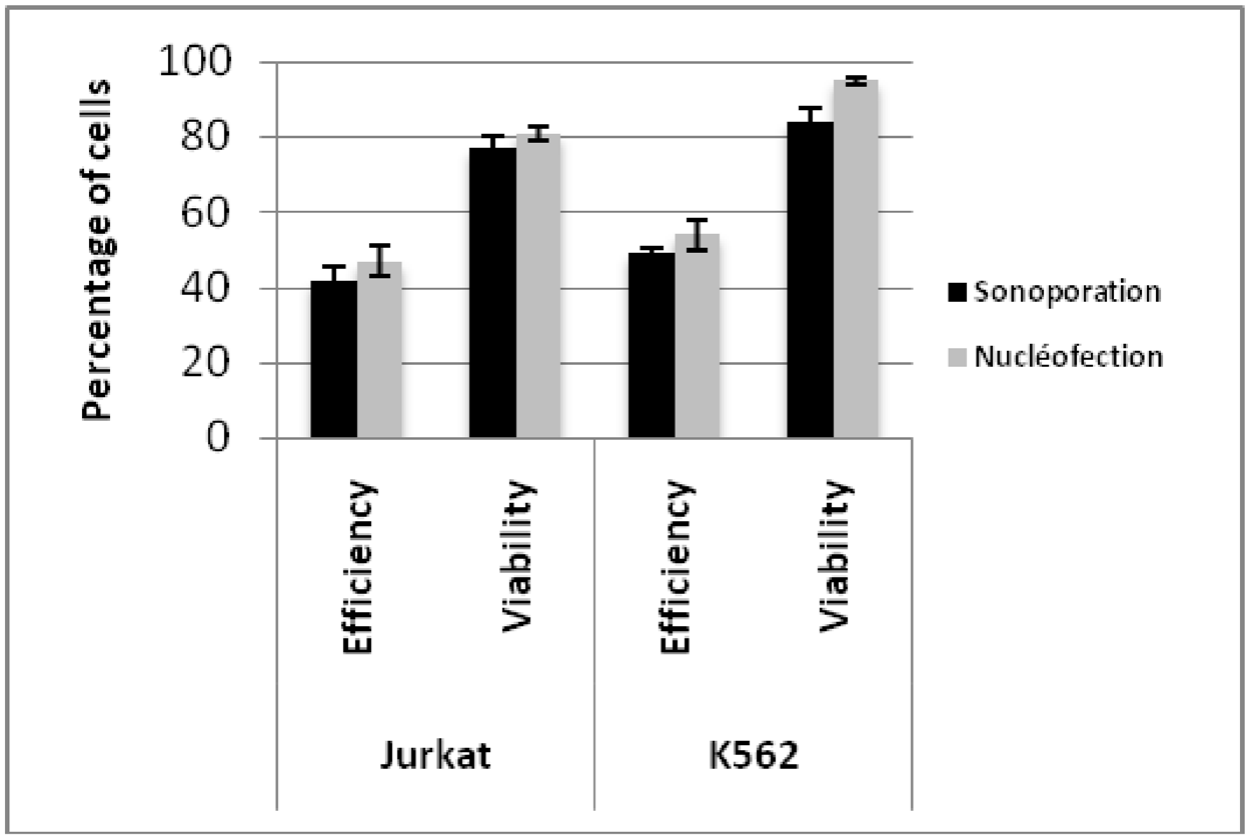
Comparison of the transfection efficacy of pEGFP-C1 with sonoporation and nucleofection. Transfection efficiency (black bar) and cell viability (grey bar) were determined by flow cytometry analysis 24 hours post-sonoporation and nucleofection of non-adherent JURKAT and K562 cell lines. The error bars represent the mean (± standard deviation, SD) of triplicate measurements.

Experiments with Jurkat and K562 cell lines were undertaken to compare the transfection efficiency of sonoporation to those of nucleofection and lipofection. Sonoporation and nucleofection were essentially equivalent in terms of DNA delivery and toxicity as determined by flow cytometry analysis and PI staining. Using sonoporation, the Jurkat line showed 77% viability and 42% transfection efficiency, while transfection of K562 gave 84% viability and 49% transfection efficiency. Nucleofection gave results essentially equivalent in terms of toxicity and efficiency (Fig 6). However, these two cell lines were resistant to transfection with lipofection methods, with less than 1% of cells transfected (data not shown).

### 3.6 Production of stably transfected cell lines

**Fig 7:**
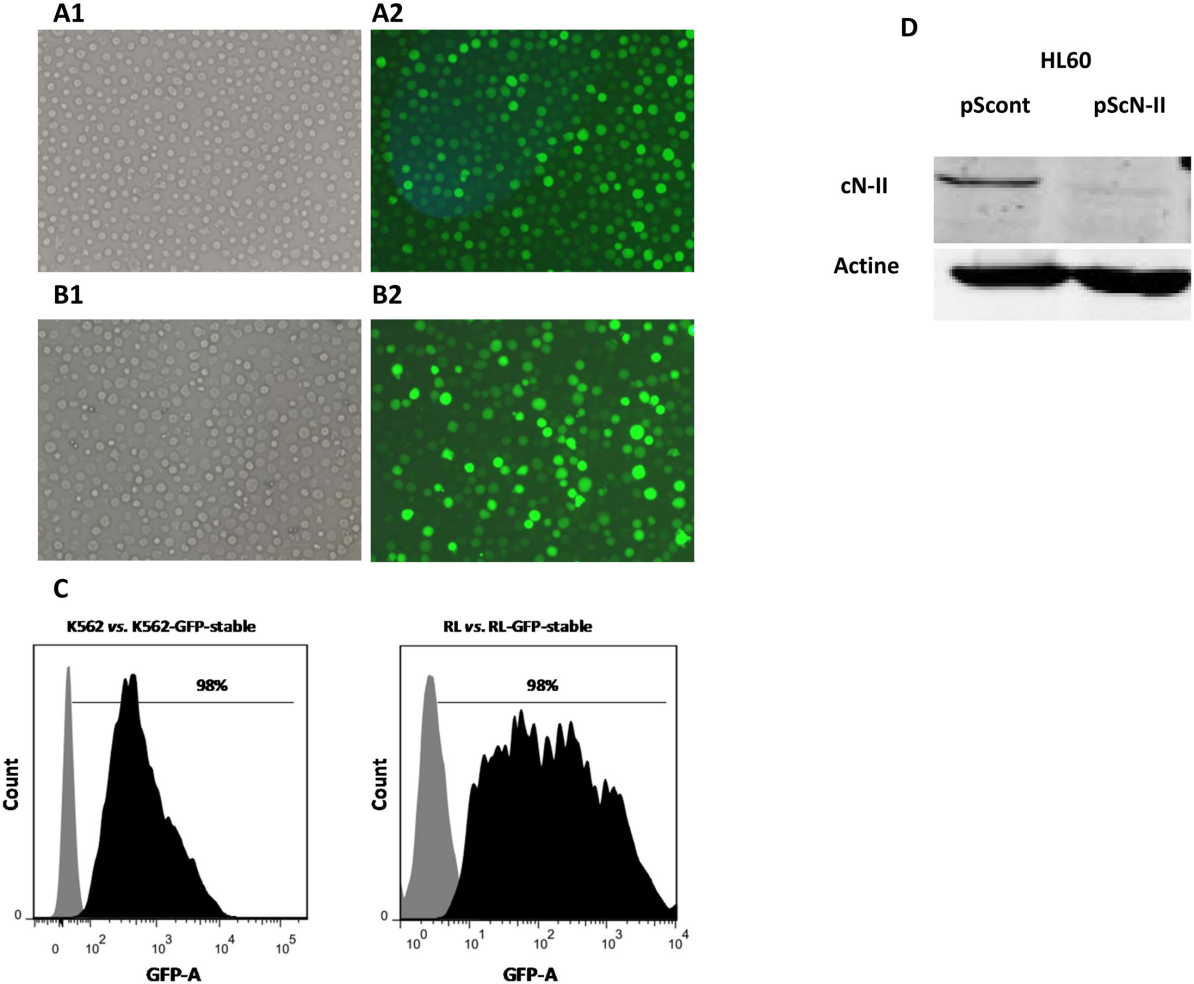
Production of stably transfected K562, RL and HL60 cells by sonoporation. K562 and RL cell lines were transfected with pEGFP-C1 plasmid and cultured in selection medium containing 1.2 and 1.5 mg/ml of G418, respectively. The microscopy pictures (objective 10×) correspond to light transmission (A1 and B1) and fluorescence images of RL-pEGFP-C1 (A2) and K562-pEGFP-C1 (B2) stable clones. (C) The GFP channel histograms obtained from flow cytometry are shown. Grey indicates non-transfected cell lines and black indicates GFP-transfected cell lines. Flow cytometry analysis revealed 98% GFP positive cells in the stable clones. (D) HL-60 cell line transfected with expression vectors coding for shRNA against cN-II (pScN-II) and control sequence (pScont) were selected with continuous exposure to 1 mg/ml (HL60-pScN-II and HL60-pScont cells) of G418. Western blot analysis clearly showed strong inhibition of cN-II expression in HL60-pScN-II cells compared with cells expressing a non-silencing shRNA. Results are representative of three independent western blot analyses.

RL and K562 cell lines were transfected with pEGFP-C1 plasmid and HL-60 cell line was transfected with vectors coding for shRNA against the cN-II nucleotidase (pScN-II) and a control scrambled sequence (pScont). Transfected cells were selected by continuous exposure to 1.2 mg/ml (RL-pEGFP-C1 cells), 1.5 mg/ml (K562-pEGFP-C1cells) and 1 mg/ml (HL60-pScN-II and HL60-pScont cells) of G418. Five to six days after the addition of selection medium the percentage of cells expressing GFP increased, representing the entire culture after 5 weeks of culture. The polyclonal cell cultures were processed using limiting dilution to isolate clones. As shown in Fig 7, flow cytometry analysis of the RL-pEGFP-C1 and K562-pEGFP-C1 clones revealed 98% GFP positive cells. cN-II protein content in HL60- pScN-II cells analyzed by western blot demonstrated a strong inhibition of cN-II protein expression in comparison with HL60-pScont cells. These results demonstrate that ultrasound mediated-transfection allows a production of stable lines from non-adherent cell lines.

## Discussion

Over the past few years, several researchers have used ultrasound-induced cavitation as a tool for drug and gene delivery. Transfer of nucleic acids or drugs is attributed to transient holes in the cell membrane produced by ultrasound-induced cavitation [19, 20]. The main problem using acoustic cavitation for transfection is the lack of stationary behavior of the initiation and leveling of cavitation which leads to uncontrolled biological effects on cells and an elevated level of cell death. The present study examined the impact of controlled cavitation on *in vitro* pDNA delivery in adherent and non-adherent cell lines using ultrasound. First, we developed a new ultrasound device wherein acoustic cavitation is stably controlled over time (Fig 2B–2D). As maintaining pDNA integrity is essential when it comes to ultrasound exposure, we demonstrated that the plasmid integrity is not affected by ultrasonic irradiation even at high CI level **(** Fig 3). On the other hand and as previously described, we demonstrated that the transfection efficiency is proportional to the level of CI [11, 17]. A CI of 12 for an exposure time of 30 seconds was considered to be optimal for Jurkat and K562 cells. Furthermore, we investigated the influence concentration on transfection efficiency in the model of suspended cells Jurkat. The results showed similar levels of transfection between the different cell concentrations tested (1.10^6^, 2.10^6^, 3.10^6^ and 10.10^6^ cells/ml) with a slight impact on technical reproducibility with densities lower than 10.10^6^ cells/ml. Thus, a CI value of 12, a 30 seconds exposure time, 10.10^6^ cells per ml and 10 μg of pEGFP-C1 are considered as the optimal transfection parameters that showed the highest transfection efficiency with minimal cell damage. The results obtained showed high transfection efficiency up to 80% with 75% of viability in the A549 lung epithelial cell line. In comparison, a sonoporation efficiency of 10–38% was previously reported on the same cell type with the combination of ultrasound and micro-bubbles [10, 28, 29]. Unlike earlier results reported by other groups [10, 11, 17, 28, 37–38], we observed high transfection efficiencies for both adherent and non-adherent cell lines using a single program, the same cell density and the same pDNA concentration.

A major advantage of our apparatus is that high transfection efficiency was obtained without using contrast agents or transfection reagents. Many studies [23–29] have emphasized the need for contrast agents to improve transfection which is relatively complicated to optimize, as well as time- and money-consuming. The data presented in Fig 6 demonstrated the reproducibility of the results obtained with our device. The cavitation device used in this study allows long-term expression of ultrasound-mediated gene transfection as demonstrated by the production of three stable hard-to-transfect non-adherent cell lines (Fig 7).

Lipofection and nucleofection are the main methods used for routine transfection of adherent and non-adherent cell lines. Lipid-mediated gene delivery methods also referred to as lipofection or liposome-based-gene transfection are easy to use but not applicable to all cell types particularly for non-adherent cell line transfection [39–40]. Nucleofection (a modified electroporation technology) is now one of the most effective non-viral methods for *in vitro* gene delivery. Electroporation-based nucleofection technique by Lonza allows high transfection efficiency in hard-to-transfect cells [41–43]. However, nucleofection relies on an expensive kit that varies from cell line to cell line. Also, neither the content of the cell-dependent electroporation buffer nor the electroporation parameters are disclosed by the manufacturer. There are concerns that the unknown additives in the buffer used for nucleofection might affect the metabolic rate of some cancer cells and can alter the subcellular distribution of the recombinant protein in some cells [43]. Experimental comparison of sonoporation to lipofection and nucleofection in transfection applications of Jurkat and K562 non-adherent cell lines allowed us to demonstrate that sonoporation and nucleofection are equivalent in terms of cell viability and transfection efficiency. Moreover, sonoporation of cells freshly isolated from human blood using our device provides higher cell viability than nucleofection [36]. Our experimental findings confirmed that lipofection is not applicable for non-adherent cell lines transfection. Sonoporation thus appears to be a potent transfection method, in particular for non-adherent cells and fresh human samples.

## Conclusion

An effective control of spatial and temporal acoustic cavitation was achieved in this study. It provides a well-adapted method for low cost routine pDNA *in vitro* delivery for both adherent and non-adherent cell lines. Our results confirm ultrasound as an alternative non-viral technology for the efficient transient transfection of a wide range of different cells including non-adherent cells or fresh human cells, and the preparation of stably transfected cells.
